# Landscape of transcription and expression regulated by DNA methylation related to age of donor and cell passage in adipose-derived mesenchymal stem cells

**DOI:** 10.18632/aging.103809

**Published:** 2020-10-31

**Authors:** Guan-Ming Lu, Yong-Xian Rong, Zhi-Jie Liang, Dong-lin Hunag, Yan-Fei Ma, Zhi-Zhai Luo, Fang-Xiao Wu, Xin-Heng Liu, Yu Liu, Steven Mo, Zhong-Quan Qi, Hong-Mian Li

**Affiliations:** 1 Department of Breast and Thyroid Surgery, Affiliated Hospital of Youjiang Medical University for Nationalities, Baise 533000, Guangxi, China; 2 Department of Burn and Plastic Surgery, Guiping People’s Hospital, Guigping 537200, Guangxi, China; 3 Department of Plastic and Aesthetic Surgery, The Fifth Affiliated Hospital of Guangxi Medical University and The First People’s Hospital of Nanning, Nanning 530022, Guangxi, China; 4 Medical College of Guangxi University, Nannig 530004, Guangxi, China; 5 Nanning Qiuzhijian Biotechnology Co., Ltd., Nanning 530229, Guangxi, China

**Keywords:** adipose-derived mesenchymal stem cells, tissue regeneration, WGCNA, regenerative medicine, TCGAbiolinks

## Abstract

Adipose-derived mesenchymal stem cells (ADSCs) are pluripotent stromal cells that can differentiate into a variety of cell types, including skin cells. High-throughput sequencing was performed on cells of different ages and cell passage, obtaining their methylation, mRNA expression, and protein profile data. The stemness of each sample was then calculated using the TCGAbiolinks package in R. Co-expression modules were identified using WGCNA, and a crosstalk analysis was performed on the corresponding modules. The ClusterProfile package was used for the functional annotation of module genes. Finally, the regulatory network diagram was visualized using the Cytoscape software. First, a total of 16 modules were identified, where 3 modules were screened that were most relevant to the phenotype. 29 genes were screened in combination of the RNA seq, DNA methylation seq and protein iTRAQ. Finally, a comprehensive landscape comprised of RNA expression, DNA methylation and protein profiles of age relevant ADSCs was constructed. Overall, the different omics of ADSCs were comprehensively analyzed in order to reveal mechanisms pertaining to their growth and development. The effects of age, cell passage, and stemness on the therapeutic effect of ADSCs were explored. Additionally, a theoretical basis for selecting appropriate ADSC donors for regenerative medicine was provided.

## INTRODUCTION

Strides in medicine have significantly improved the level of wound repair, but the functional and cosmetic burden that occur following wound healing remains problematic [1]. In recent years, research has suggested the feasibility of using stem cells in clinical post-traumatic repair and have potentially surprising therapeutic efficacy [2, 3]. Although highly beneficial in theory, due to certain regulatory and ethical considerations, embryonic stem cells (ESC) and induced pluripotent stem cells (iPSC) are restricted in clinical applications [4, 5]. Compared to ESC, adipose-derived mesenchymal stem cells (ADSCs) are more ideal stem cell populations in the field of regenerative medicine. ADSCs may be more easily obtained without ethical concerns [6, 7]. ADSCs were first isolated and extracted from adipose tissue by Zuk and colleagues [2]. ADSCs are ideal in many ways: they can be harvested, processed, and propagated non-invasively, easily and efficiently. Their pluripotency and proliferation efficiency are not lower than those of bone marrow-derived MSCs, and the incidence of donors was lower than MSC collected from other sites [2]. With the development of medical technology, ADSCs have been used in the treatment of diabetic foot [8], joint degenerative disease repair [9], bone repair [10], breast reconstruction [11], and ischemic disease [12]. Animal and clinical studies have shown that ADSCs can repair damaged bone tissue or large bone segmental defects. [13, 14].

Researchers have also discovered that the epigenetic environment affects the regeneration ability of human ASCs [15]. In clinical practice, we found that the therapeutic effect of ADSCs is closely related to the age of the donor, number of passages of the cells, and stemness of the cells. However, it is unclear how such factors affect the growth and development of ADSCs. In order to better understand the mechanism of ADSCs’ growth and development, a high-throughput sequencing analysis of ADSCs of different ages and varying cell passages was performed in this study. Moreover, 3 modules were identified with the most significant phenotypic correlation from 16 WGCNA modules. In addition, long non-coding RNA (lncRNA) and transcription factors (TFs) were identified that could regulate the genes of the WGCNA module. Finally, a functional enrichment analysis was conducted for the module genes, and a global regulatory network was constructed in order to comprehensively explain the molecular mechanism of ADSC development.

## RESULTS

**Figure 1:**
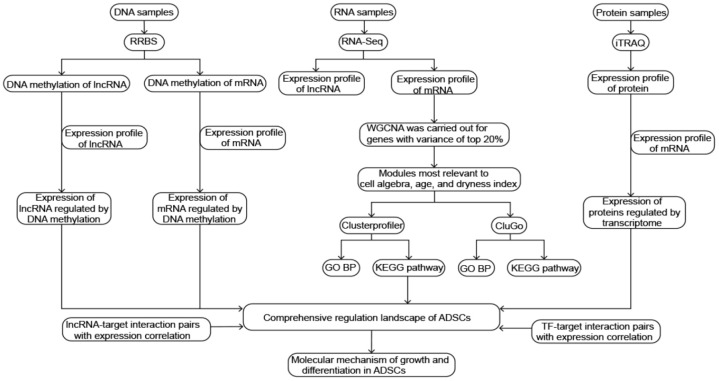
**Flowchart in this study.**

The steps of the present study are detailed in Figure 1.

### Characteristics of ADSCs in different patient ages and cell passages

**Figure 2:**
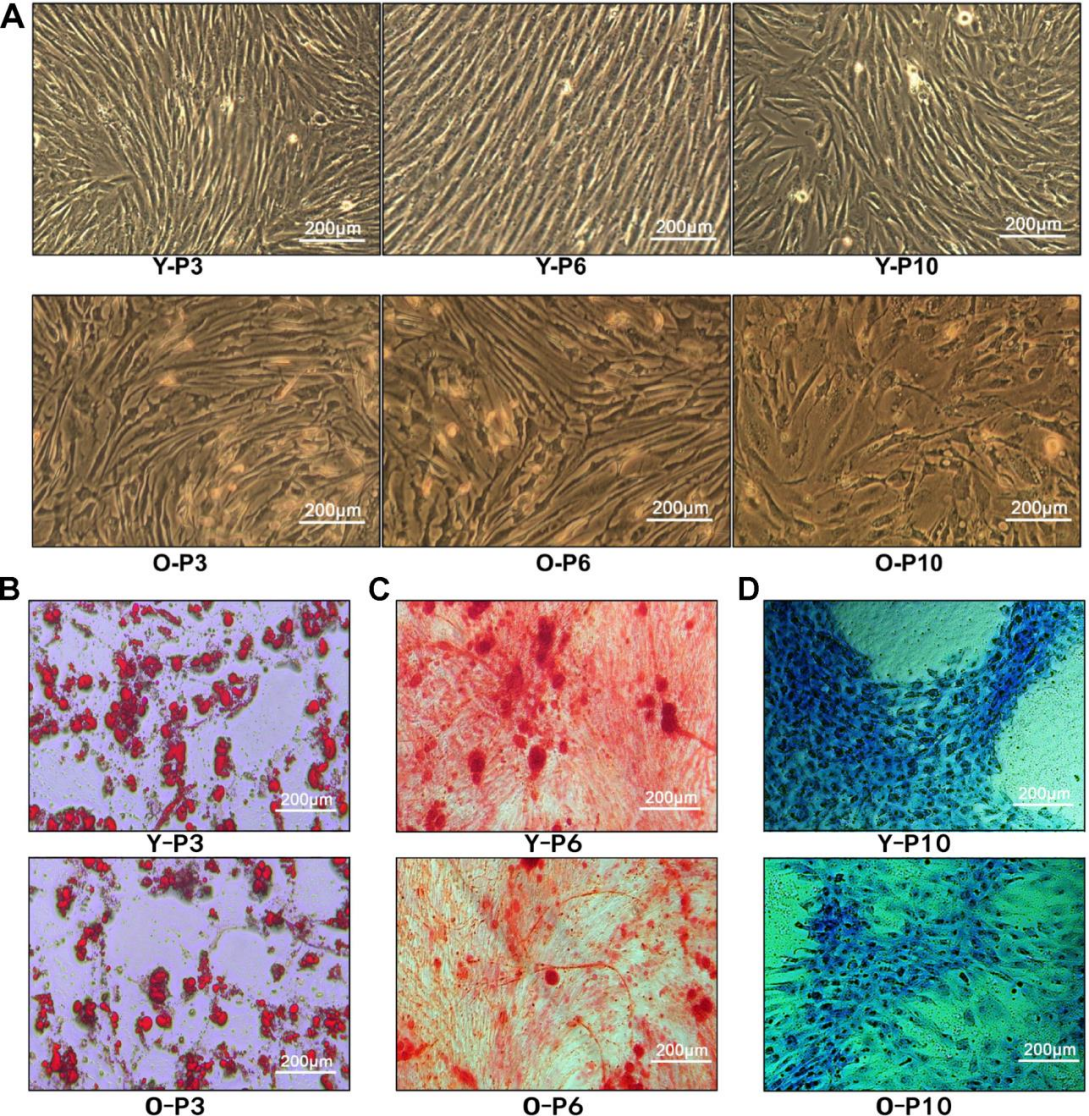
**Characterization of ADSCs.** (**A**) 3^rd^, 6^th^, and 10^th^ passage Spindle-shaped ADSCs in young and old donors. Y-P3, Y-P6, Y-P10 represent the 3^rd^, 6^th^, and 10^th^ passages of the young donor, respectively. O-P3, O-P6, O-P10 represent the 3^rd^, 6^th^, and 10^th^ passages of the old donor, respectively. (**B**) Micrographs of 3^rd^ generation ADSCs of young patients (up) and old patients (down) stained with Oil Red O. (**C**) Micrographs of 3^rd^ generation ADSCs of young patients (up) and old patients (down) stained with Alizarin Red. (**D**) Micrographs of 3^rd^ generation ADSCs of young patients (up) and old patients (down) stained with Alcian Blue.

ADSCs were extracted from fat extracts, separated and amplified. Homogeneous ADSCs were observed until they reached 80–90% confluency at 7–14 days. The cells grew in a monolayer with spindle-shaped morphologies and exhibited strong proliferation. Both the 6^th^ and 10^th^ passages of ADSCs developed into normal morphology (Figure 2A), indicating that treatment with ADSCs may not cause malignant transformation. In addition, the proliferative capacity of ADSCs was found to be stronger in the young group than in the elderly group, indicating that the age of the donor is an important factor affecting ADSCs’ proliferation.

ADSCs of the third passage in adipogenic, osteogenic, and chondrogenic media were cultured for 2 or 3 weeks. Cell morphologies were observed during the specific induction of each lineage. Multipotency of ADSCs was verified by positive staining for oil red O (Figure 2B), alizarin red (Figure 2C), and alcian blue (Figure 2D), demonstrating adipogenic, osteogenic, and chondrogenic differentiation, respectively. Consequently, the results demonstrated that ADSCs have the potential to differentiate into adipocytes, osteoblasts, and chondrocytes. Regardless of adipogenic, chondrogenic or osteoblastic differentiation, compared to the old group, the young group received more cell numbers, indicating that the younger group had better cell differentiation ability.

### Identifying phenotype-related gene co-expression modules

**Figure 3:**
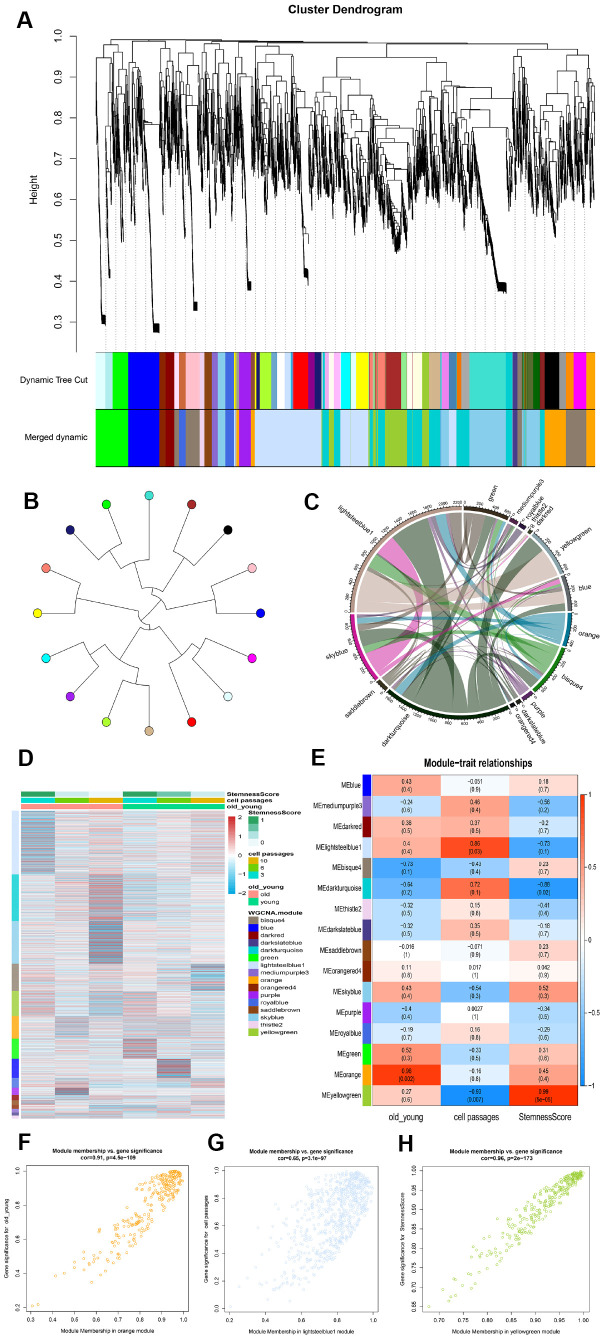
**Identification of modules that was significantly correlated with phenotype.** (**A**) Clustering dendrogram and modules. Each gene is represented by a leaf in the tree, where the y-axis represents the network distance determined by the topological overlap (TO) and different colors indicate the combined module membership. A total of 16 modules were identified. (**B**) Module tree diagram. Each point represents a module, and the lines indicate the interaction between the modules. (**C**) Circplot between modules. (**D**) Cluster analysis heatmap showing the expression of different modules in different phenotypes. (**E**) A heatmap of the correlation between module eigengenes and ADSCs phenotype. (**F**–**H**) The scatter plot shows the correlation between different phenotypes and MEorange, MElightsteelblue1 and MEyellowgreen.

In order to ascertain the key module most associated with the age and passages of ADSCs, WGCNA was performed using the expression profile of the mRNA. The power was 13, which was the lowest value for the scale with an independence degree of up to 0.80. The co-expression network contained 16 modules (Figure 3A). Next, a tree diagram was constructed for the 16 modules based on the co-expression clustering distance, showing the correlation between each module (Figure 3B). In addition, a Crosstalk analysis was performed on the module, where a total of 54 Crosstalk, including 4260 interaction pairs, were obtained (Figure 3C).

Subsequently, the stemness of each sample was calculated according to the mRNA expression profiles, after which a module expression heat map was constructed (Figure 3D). The samples were then divided into three phenotypes: age, cell passages and stemness. As the number of cell passages increased, the stemness also appeared to decrease accordingly. Compared to the old group cells, the young group displayed higher stemness. The correlation and significance between the modules and phenotypes were also calculated. The orange module was found to be the module most associated with the age phenotype (cor=0.96); the lightsteelblue1 module was the module most associated with the cell passages phenotype (cor=0.86); and the yellowgreen module was the module most associated with the stemness phenotype (cor=0.99) (Figure 3E). A scatter plot showing the linear correlation between phenotype and module was then established (Figures 3F–3H).

### Module-related biological processes and pathways

**Figure 4:**
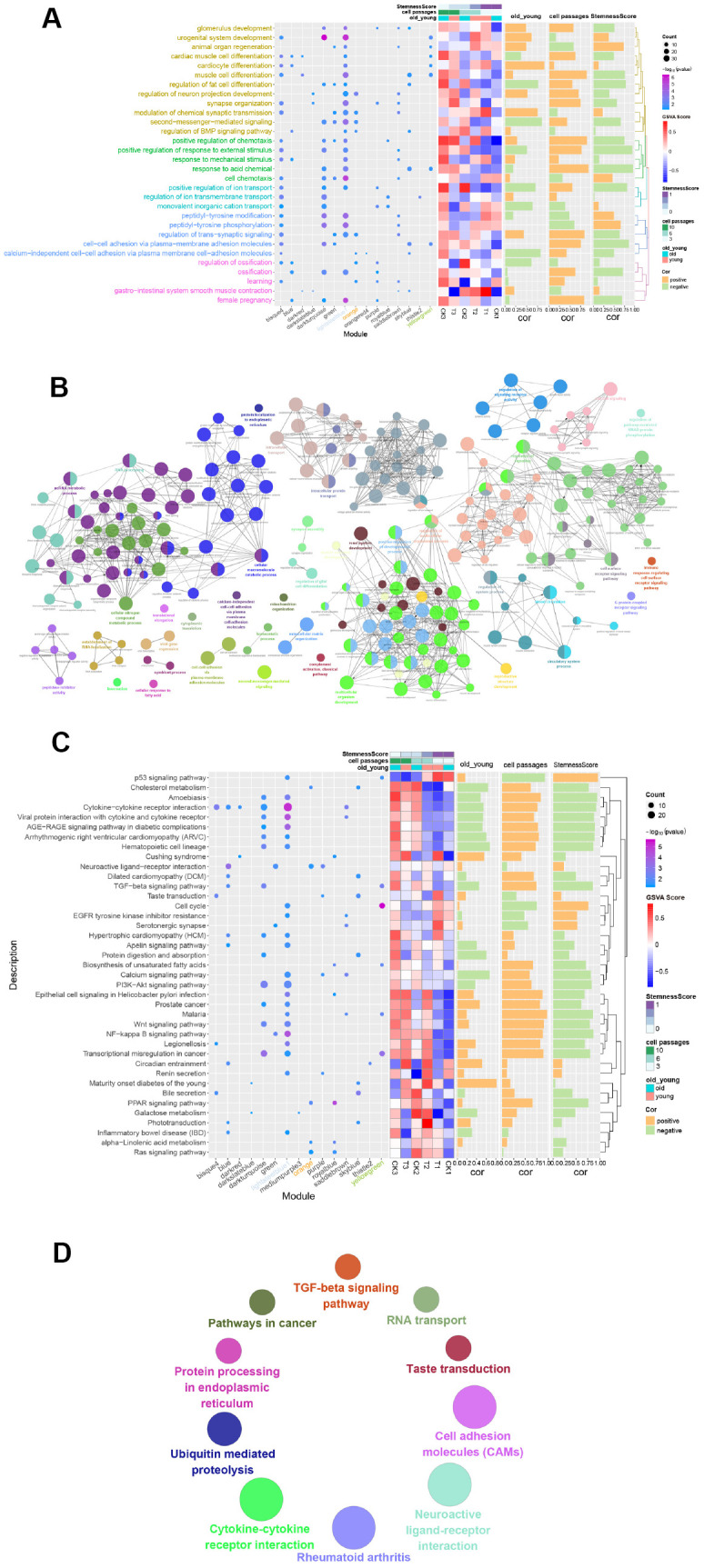
**Functional enrichment analysis of gene modules.** (**A**) GO Enrichment Analysis. Enrichment increased significantly from blue to purple. The larger the circle, the more significant the percentage of module genes that GO functions into the gene. (**B**) Gene functional enrichment was conducted using Cytoscape. Each dot represents a BP, and a total of 244 are enriched. (**C**) Enrichment Analysis of the Module Gene KEGG Pathway. From blue to purple, the concentration increased significantly. The larger the circle, the more significant the proportion of modular genes present in the KEGG pathway entry genes. (**D**) Enriched to 10 KEGG pathways using the ClueGO plugin for Cytoscape.

Figure 4A shows GO_BP terms, which was found to significantly enrich more than the four modules. The genes of the lightsteelblue1 and yellowgreen modules were significantly enriched in “urogenital system development” and “muscle cell differentiation”. The genes of the orange and lightsteelblue1 modules were significantly enriched in “regulation of neuron projection development” and “second-messenger-mediated signaling”. In addition, a CluGO network was built, where a total of 10 functions overlapped with the above 30 functions (Figure 4B).

By performing a KEGG analysis on the 16 modules of genes, 39 pathways with the highest number of participations were obtained (number of times≥2) (Figure 4C). The results showed that genes of the lightsteelblue1 module were significantly enriched in the “Cytokine-cytokine receptor interaction pathway”, genes of the yellowgreen module were significantly enriched in the “Cell cycle pathway”, and genes in the orange module were significantly enriched in the “Neuroactive ligand-receptor interaction pathway”. Similarly, the ClueGO network of the KEGG pathways (Figure 4D) were constructed. Here, a total of 4 pathways overlapped with the 39 KEGG pathways mentioned above, namely, “Cytokine-cytokine receptor interaction”, “Neuroactive ligand-receptor interaction”, “Taste transduction” and “TGF-beta signaling pathway”.

### Methylation regulates gene expression in ADSCs

**Figure 5:**
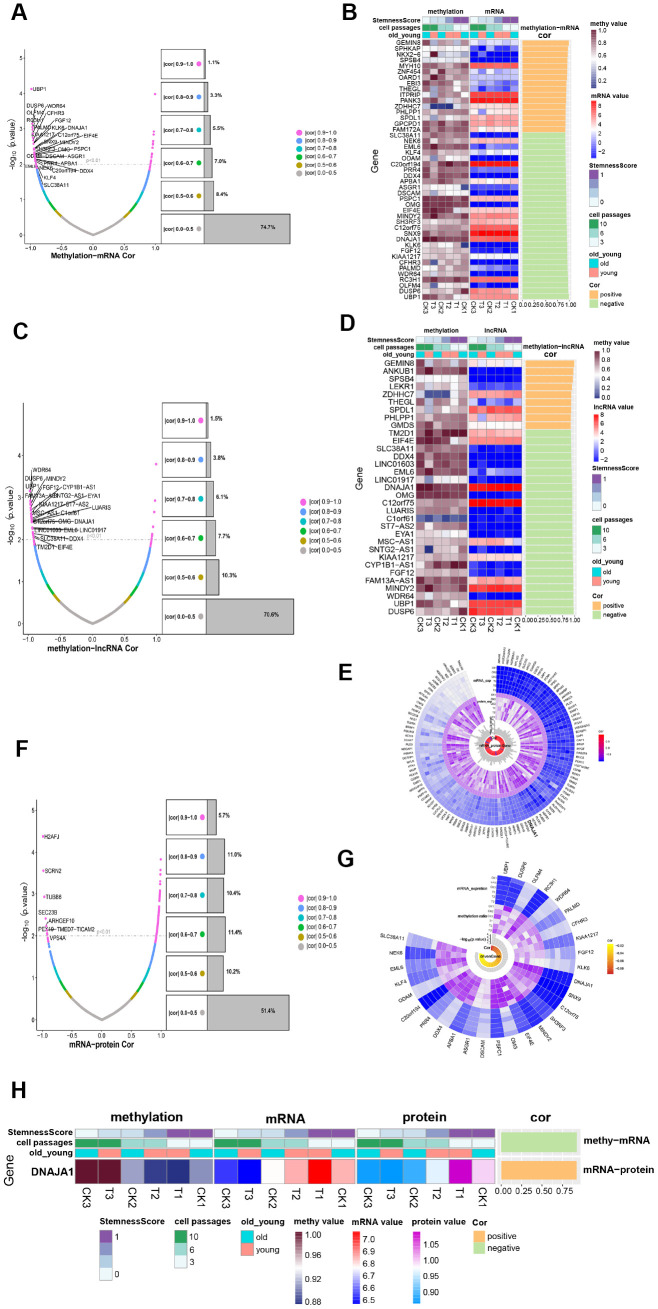
**Methylation regulates ADSCs’ expression.** (**A**) Volcanic maps show the correlation and significance of methylation and mRNA expression. (**B**) Heatmap shows the expression of methylation-transcription related genes among different phenotypes. (**C**) Volcanic maps show correlation and significance of methylation and lncRNA expression. (**D**) Heatmap shows the expression of methylation-lncRNA related genes in different phenotypes. (**E**) CircPlot shows the methylation rate and mRNA expression of 29 methylation-regulated transcription genes in 6 samples as well as their correlation and significance. (**F**) Volcanic maps show the correlation and significance of mRNA and protein expression. (**G**) circPlot displays the mRNA and protein expression of 117 mRNA_protein related genes in 6 samples, and their correlation and significance, highlighting a methylation-regulated expression gene. (**H**) Heat Map-Correlation Histogram showing DNAJA1 expression.

To obtain insight into the development of ADSCs, the methylation profile was combined with the mRNA expression profile as well as the lncRNA expression profile, respectively. The methylation profile was compared to the mRNA expression profile to intersect, where a total of 5427 genes were obtained. Figure 5A shows the correlation and significance of methylation and mRNA expression. Among the 5427 genes where methylation and mRNA intersected, 45 significantly related genes were identified (|cor|>0.9, P<0.01) (Figure 5B) called methylation transcription-related genes. The methylation profile was compared to the lncRNA expression profile, and a total of 4308 intersection genes were obtained (Figure 5C). Among the 4308 genes where methylation intersected with lncRNA, 33 significantly related genes were identified (|cor|>0.9, P<0.01) (Figure 5D), of which 19 were methylation-regulated transcription genes. Then, 29 genes that had a negative correlation between methylation and mRNA expression were selected, which were considered to be transcription genes regulated by methylation. As shown in Figure 5E, most of these 29 methylation-regulated transcripts demonstrated formidable significance and correlation among the 6 samples. Of these genes, UBP1 exhibited the most significant correlation.

After combining the mRNA expression profile with the protein expression profile and comparing their intersections, 2874 intersection genes were yielded, of which 117 were significantly related genes (Figure 5F). As shown in Figure 5G, these genes were highly expressed in all 6 samples and possessed significant correlations. By comparing the 117 transcriptionally regulated proteins with 29 methylation-regulated transcription genes, a methylation-regulated expression gene DNAJA1 was obtained (Figure 5H). DNAJA1 has been shown to be related to immune responses [16], and its role in the development of ADSCs deserves further investigation.

### Comprehensive regulation landscape of ADSCs

**Figure 6:**
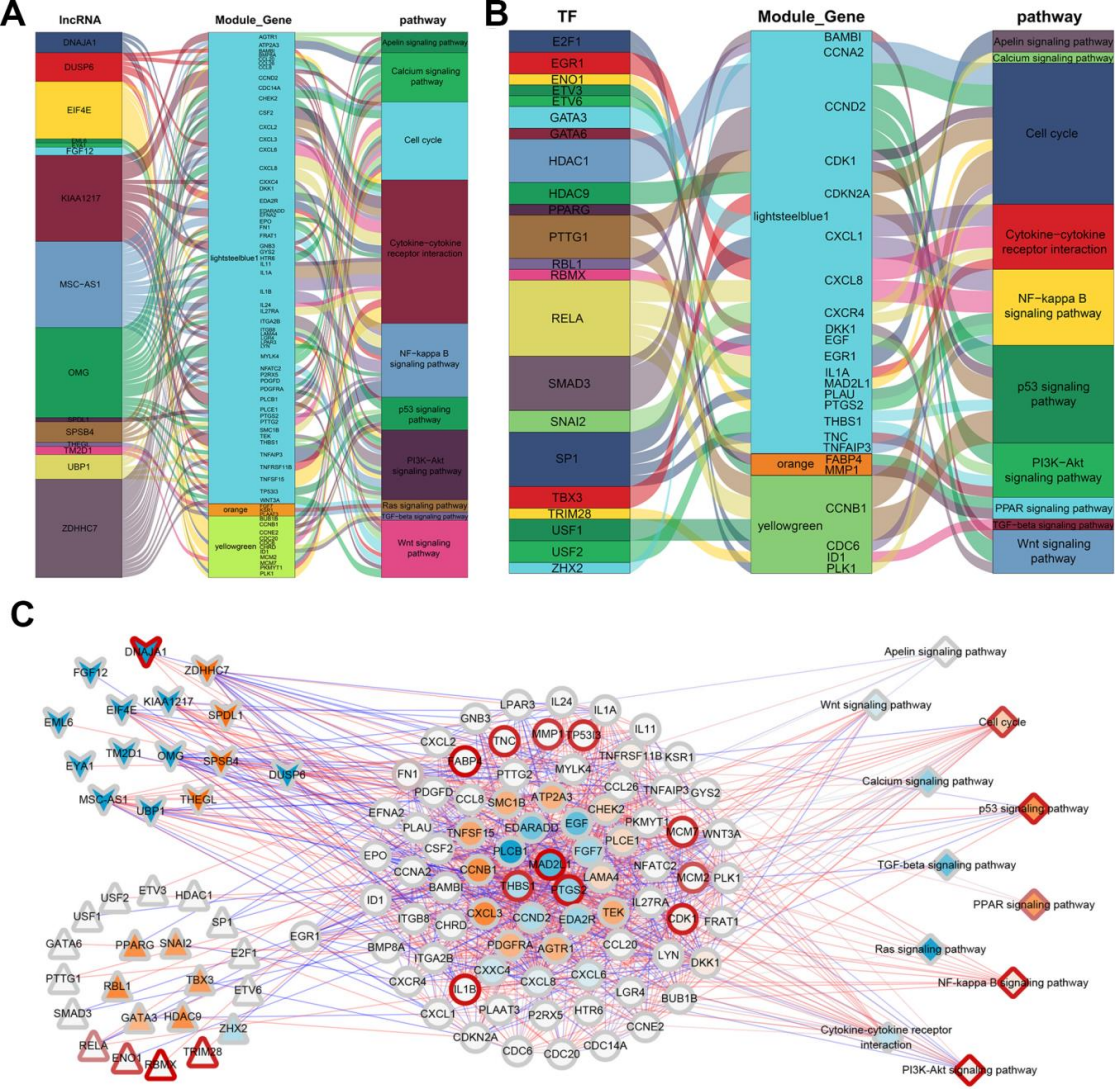
**ADSCs’ integrated regulatory network.** (**A**) Sankey plot of gene module regulation by lncRNAs. Each square on the left represents a lncRNA, where the middle square represents a modular gene and the right square represents the pathway. (**B**) Map of gene module regulation by TFs. Each square on the left represents a TF, where the middle square represents a modular gene and the right square represents a pathway. (**C**) Integrated regulatory network of lncRNA/TF-target genes-pathways. Squares represent pathways; circles represent modular genes; arrows represent lncRNA; and triangles represent TFs.

To illustrate the relationship between the identified genes and the three phenotypic modules, a correlation analysis was performed between the 33 methylation-regulated lncRNA and the 1394 module genes. After combining the lncRNA expression profile and the mRNA expression profile, genes having a significant correlation (|cor|>0.9, p<0.01) were selected and compared to the module genes, and 19 lncRNA involved in the regulation of the three most significant modules were obtained. Interestingly, one of the genes, DUSP6, was observed to be both a lncRNA and module gene. According to Fan et al, DUSP6 can inhibit epithelial-mesenchymal transition [17]. The 19 lncRNA were then compared to the previously enriched genes involved in the KEGG pathway, which showed that 15 lncRNA participated in regulating 27 KEGG pathways through 92 module genes. Ten pathways related to ADSCs were selected to construct a regulatory network map. As shown in Figure 6A, 15 lncRNA participated in the regulation of 10 KEGG pathways through 68 module genes.

Based on the interaction of human transcription factors (TFs) and their target genes in the TRRUST v2 [18] database, a correlation analysis was conducted on the interaction pairs of TFs and target genes. Here, 440 significantly related gene pairs (|cor|>0.9, p<0.01) intersected with the module genes, and 40 TFs involved in the regulation of the three most significant modules were obtained. The intersection of these 40 TFs and genes involved in the KEGG pathway showed that 24 TFs participated in regulating 21 KEGG pathways through 29 module genes. Similarly, 10 pathways related to ADSCs were selected so as to construct a regulatory network map. The results demonstrated that 22 TFs participated in the regulation of 10 KEGG pathways through 24 module genes (Figure 6B).

**Figure 7:**
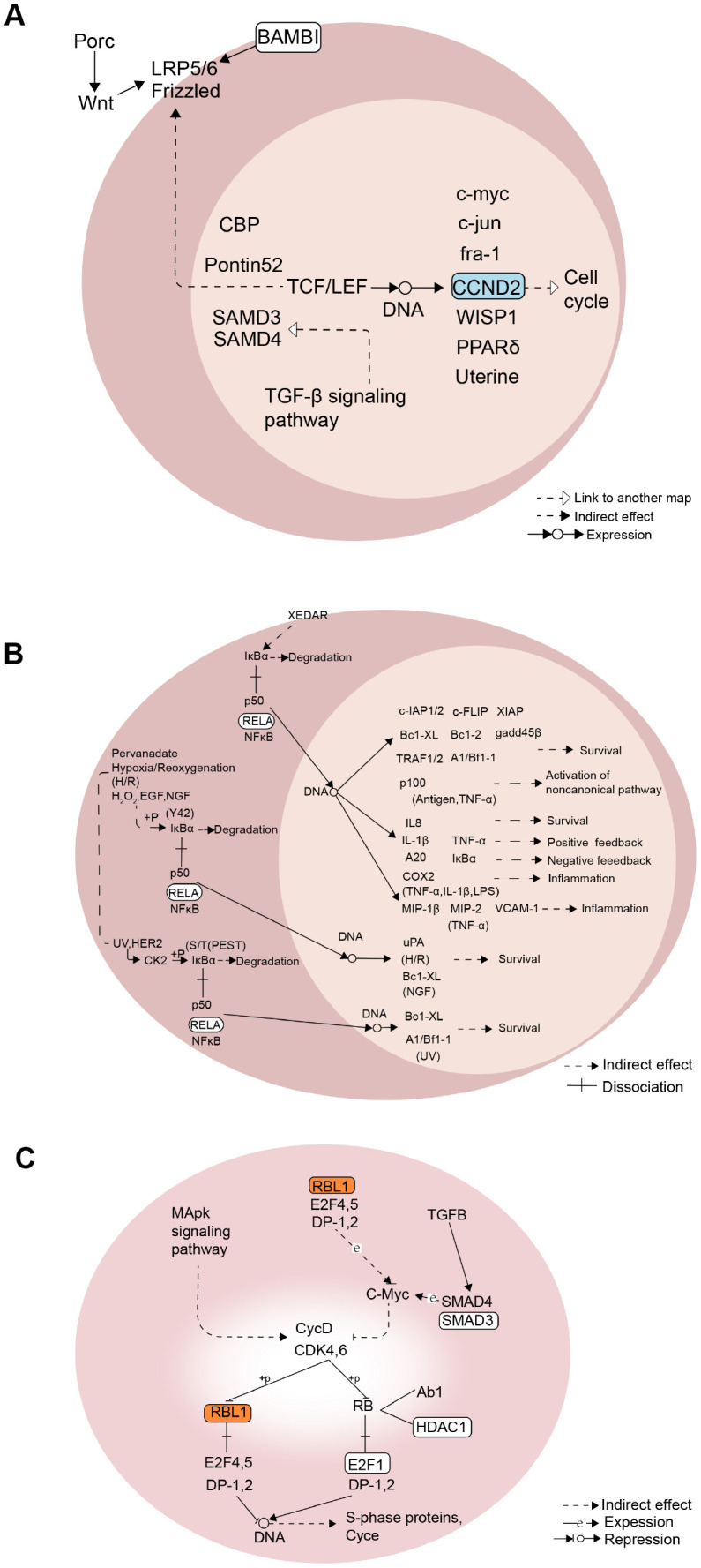
**Mechanism diagram of the KEGG pathway.** (**A**) Wnt signaling pathway. (**B**) NF-kappa B signaling pathway. (**C**) Cell cycle pathway.

According to the GSVA scores of the 39 KEGG pathways in the methylation, mRNA expression, lncRNA and protein expression profiles, the correlation between methylation and RNA or RNA and protein were analyzed. Finally, a comprehensive regulatory network was built using the Cytoscape [19] software. In this network, 15 lncRNA and 22 TFs participated in the regulation of 11 KEGG pathways through 80 module genes (Figure 6C). Overall, the 11 screened KEGG pathways were found to play an important role in the growth and development of ADSCs. Mechanically, in the Wnt signaling pathway, the expression of CCND2 was regulated by TCF / LEF, which affects the cell cycle (Figure 7A). RELA affects the survival and inflammation of cells through the NF-kappa B signaling pathway. In addition, it can also lead to the activation of noncanonical pathway of cells (Figure 7B). In the Cell cycle pathway, RBL1 can affect the expression of C-Myc, indirectly affecting S-phase proteins (Figure 7C).

## DISCUSSION

Due to the continuous advancement of medical technology, the scope of ADSCs’ application has widened. The potential of ADSCs for use in regenerative medicine has been extensively explored. ADSCs are multipotent stromal cells that differentiate into a variety of cell types [20]. Studies have shown that ADSCs may successfully differentiate into a neural lineage and Schwann cells [21, 22]. Many studies have confirmed that ADSCs play an indispensable role in regenerative medicine [23, 24]. Furthermore, research has demonstrated that the use of ADSCs is safe and feasible in the clinical treatment of wounds [25, 26].

To ensure the effectiveness and safety of ADSC transplantation, the choice of donor is particularly important. Age, cell passage and stemness largely determine the quality of ADSC donors. However, phenotype-related genes that affect the therapeutic effect of ADSCs, as well as the molecular mechanisms that occur in the course of regenerative medicine, remain unclear. Therefore, from the perspective of multiomics, the molecular mechanism of ADSCs at different ages and different cell passage was comprehensively analyzed in the present study. It is commonly thought that the younger the donor is, the less the passage of cells and the higher the stemness of cells. Nevertheless, this study found that even in an old group of ADSCs, when the number of cell passages is small, the stemness is also high, indicating that cell passage has a greater effect on the stemness of ADSCs than donor age.

In order to explore this mechanism, the modules most relevant to the three phenotypes were identified: MEorange, MElightsteelblue1, and MEyellowgreen. In addition, function and pathway enrichment analyses were also performed on the modules. The enrichment analysis showed that functional modules involve multiple GO terms and pathways, which may likely reflect the complexity of ADSCs. Moreover, MElightsteelblue1 (the module most relevant to the cell passage) was found to be significantly enriched in “Apelin signaling pathway”. Apelin is the endogenous ligand for APJ, which is widely distributed in the limbs, heart, brain, adipose tissue, and kidney. Studies have found that Apelin regulates the osteogenic differentiation of mesenchymal stem cells through the "Wnt / β-catenin signaling pathway" and promotes bone healing [27]. By conducting an in-depth analysis of methylation, transcription expression and protein profiles, 29 methylation-regulated transcription genes, 33 methylation-regulated lncRNAs, and 117 transcription-regulated proteins were identified. Particularly, among the methylation-regulated transcription-related genes, UBP1 was found to be the most relevant gene. Upstream-binding protein 1 (UBP1) is a transcriptional activator that operates in a promoter context-dependent manner. Previous studies have shown that UBP1 is closely related to biological processes like "angiogenesis" and "Transcription regulation" [28], indicating that UBP1 may play an important regulatory role in the value-added differentiation of ADSCs. In addition, the gene DNAJA1 was identified, which intersected with methylation, transcription, and protein and was defined as a methylation-regulated expression gene. Studies have found that the fate of misfolded mutp53 is regulated by DNAJA1. Inhibition of DNAJA1 induces CHIP-mediated mutp53 degradation, while its overexpression antagonizes statin-induced mutp53 degradation [29]. Jaime et al have demonstrated that the overexpression of DNAJA1 reduces the viability of pancreatic cancer cells [30]. Whether the presence of DNAJA1 may also reduce cell mutations after ADSC transplantation warrants further investigation.

ADSCs are considered to have stronger immunosuppressive effects than mesenchymal stem cells (MSCs) from different tissue sources [31] and possess clinical potential for the treatment of immune diseases [32]. The immunosuppressive capacity of ASCs is both dose-dependent and cell-passaging [33]. In this study, the transcription factor RELA was found to regulate the NF-κB signaling pathway by regulating the genes of lightsteelblue1 modules, such as CXCL8 and TNFAIP3. The NF-κB signaling pathway is closely related to immune and inflammatory responses, and the abnormal activation of this pathway is involved in the pathogenesis of various autoimmune and inflammatory diseases [34], indicating that the number of passages of ADSCs is also related to immunomodulatory pathways.

Finally, a comprehensive regulatory network was innovatively built in the present study. Accordingly, 15 lncRNA and 22 TFs were involved in regulating 11 KEGG pathways through 80 module genes. These genes are considered to be important genes in the growth and development of ADSCs, and these pathways serve as molecular mechanisms of their role. In summary, in this regulatory network, the identified genes regulate different pathways through varying molecular mechanisms, thereby affecting the growth and development of ADSCs.

Certain limitations are present in this study. First, only two donors were enrolled in the study. Although thousands of genes were obtained by sequencing, the relatively small number of patients may have reduced the reliability of the conclusions. Second, this study is limited to computer simulations, hence, the findings should be validated and extended in laboratory experiments. Although this investigation showed that lncRNA and TFs play important roles in the growth and development of ADSCs, further experiments should confirm the molecular processes in which they are involved.

## MATERIALS AND METHODS

### Donors and samples

This study was approved by the ethics committee of the First People's Hospital of Nanning, Guangxi Zhuang Autonomous Region. Written informed consent was obtained from all donors. Human lipoaspirate from abdominal subcutaneous fat from healthy adult donors (Table 1) was stored for less than 48 hr at 4°C before processing. The ADSCs of human lipoaspirate was obtained by type I collagenase digestion. Briefly, lipoaspirate was diluted with an equal volume of phosphate-buffered saline (PBS) and digested with Dulbecco's modified Eagle's medium (DMEM) containing 10% bovine serum albumin and 1 mg/ml type I collagenase for 1 hr under agitation at 37°C. The isolated cells were seeded into cell culture dishes and incubated at 37 °C in 5% CO2. The cells were then observed under a microscope to evaluate their expansion rate and cell morphology, harvested at a fusion degree of 80% to 90%, and passaged at a ratio of 1:3. Cells were taken from passages 3, 6, and 10 for later genome methylation sequencing, RNA sequencing (RNA-seq), and mass spectrum (MS) analysis. Finally, the 3^rd^, 6^th^, and 10^th^ passages cells of the young and old donors were collected, respectively, for a total of 6 samples.

**Table 1:** Subject characteristics.

**Subject**	**Age(yr)**	**Sex**	**Height (cm)**	**Weight (kg)**	**BMI (body mass index)**	**health condition**
S1	24	F	161	53	20.4	health
S2	62	F	160	55	21.5	health

### Cell-induced differentiation and staining

### Adipogenic induction

ADSCs were cultured in human adipose-derived stem cell adipogenic differentiation basal medium containing fetal bovine serum (20mL), glutamine (2mL), penicillin-streptomycin (2mL), insulin (400uL), IBMX (200uL), rosiglitazone (200uL) and dexamethasone (200uL) for two weeks. Cells were then placed in a 37 °C, 5% CO_2_ incubator, and after adipogenic differentiation was completed, the cells were stained with Oil Red O for 30 minutes and washed with 1 × PBS 2-3 times. The effect of adipogenic staining was observed under a microscope.

### Osteogenesis induction

ADSCs were cultured in human adipose-derived stem cell osteogenic differentiation basal medium containing fetal bovine serum (20mL), penicillin-streptomycin (2mL), glutamine (2mL), ascorbate (400uL), β-glycerophosphate (2mL) and dexamethasone (20uL) for two weeks. After osteogenic differentiation was completed, a staining analysis was performed using alizarin red staining solution. The culture plate was placed under a microscope to observe the effect of osteogenic staining.

### Chondrogenic induction

ADSCs were cultured in human adipose-derived stem cell chondrogenic differentiation basal medium containing dexamethasone (20uL), ascorbate (600uL), ITS + supplement (2mL), sodium pyruvate (200uL), proline (200uL) and TGF-β3 (2mL) for two weeks. After chondrogenic induction was completed, the cartilage ball was embedded in paraffin, sectioned, and stained with alixin blue. The effect of Alisin blue staining was then observed under a microscope. The partially stained Alisin blue stain demonstrated endo-acid mucopolysaccharide in cartilage tissue.

### RNA extraction and sequencing

Total RNA was isolated using the RNeasy Micro Kit, after which rRNA was removed and all non-coding RNA (ncRNA) were retained to the maximum. The obtained mRNA was randomly interrupted into short fragments, and the fragmented mRNA was used as a template to synthesize the first strand of cDNA with random hexamers. Buffers, dNTPs, RNase H, and DNA polymerase I were then added to synthesize the second strand of cDNA. QiaQuick PCR kit was used for purification, and EB buffer was added to elute the terminal repair. Next, base A and sequencing adapter was added, after which the second chain was degraded by Uracil-N-Glycosylase. Bru-labeled, nascent RNA was then isolated, converted into cDNA libraries and sequenced using an Illumina HiSeqTM 2500 sequencer. The raw data obtained following sequencing was first filtered to remove low quality data and adapters to obtain HQ clean data. The reads of the filtered rRNA were compared to the reference genome, which was followed by transcript reconstruction. The assembled transcript was compared to known mRNA and lncRNA reference sequences so as to obtain all novel transcripts. Following the prediction of coding potential, the coding and non-coding parts were distinguished in order to obtain the final predicted lncRNA. The raw image data obtained by sequencing was converted into sequence data via base calling. It was then termed raw data, after which the results were stored in the FASTQ file format.

### DNA methylation profile

LCM-DNAs from ADSCs were fragmented to 100–500 bp by 44 psi of gas for 1 min through a nebulizer and subjected to methylated DNA enrichment using a MethylMiner Methylated DNA Enrichment Kit. Then, the methylated fragments were eluted with High-Salt Elution Buffer (Invitrogen) and purified with a MinElute PCR Purification Kit (Qiagen). The DNA samples were end-repaired, ligated to Illumina methylated DNA adaptors and separated on agarose gel to isolate 150 ~ 340 bp fragments. DNA was then bisulfite converted using ZYMO EZ DNA Methylation-Gold kit and amplified by PCR. Each RRBS (Reduced representation bisulfite sequencing) library was sequenced on the Illumina platform Hisise PE150 sequencer. The bismark tool was utilized to compare the available data with the reference genome to acquire the comparison results. Finally, a methylation analysis was performed using methylKit.

### Proteome sequencing

Isobaric Tags for Relative and Absolute Quantitation (iTRAQ) technology is a proteomic quantification technology that can perform up to 8 samples in one experiment. This form of quantification can quantify almost any protein sample and possesses characteristics of high quantitative precision, which has been widely used in quantitative proteomics [35]. First, the protein was extracted from the sample and subjected to reduction and alkylation, after which the proteins in each group were digested overnight with trypsin (4.44 μg/10 μL) at 37°C. Samples were then labelled with iTRAQ reagents by following the protocol provided by the vendor. iTRAQ labelled peptides were fractionated with strong cation exchange C18 SPE, dried under a vacuum and passed through a C18 SPE cartridge to desalt the sample. It was then eluted with 500 μl CH3CN : water : formic acid (50:50:0.1, v:v:v), followed by 200 μl CH3CN. The flow-through was then passed through an Oasis C18 SPE cartridge to maximise sample recovery by capturing any peptides that may have passed through the C18 SPE.

The iTRAQ labeled peptides were subsequently dried, reconstituted and acidified with 10 mM monopotassium phosphate and 25% acetonitrile (pH 3.0) for further fractionation by strong cation exchange (SCX) chromatography. The protein concentrations of all eluted samples were monitored by an absorbance at 214 nm, and fractions were collected every minute. The collected fractions were subsequently desalted on C18 Cartridges and concentrated via vacuum centrifugation. All samples were stored at −80 °C for Liquid Chromatography Coupled with Tandem Mass Spectrometry (LC/MS/MS) analysis. Peak identification was performed on the original file obtained after mass spectrometry analysis in order to obtain a peak list. A reference database was established to identify peptides and proteins, and the relative content of each protein in each sample was compared to obtain the protein profile data.

### Data preprocessing

The sequencing data were normalized using the “*voom*” function of the limma package [36] in R, which obtained a total of 19,189 genes and 21,965 lncRNAs. Proteome and methylation sequencing data did not need to be normalized. Hence, a total of 3025 proteins and 8070 methylated genes were obtained.

### Stemness calculation

Stemness is considered to be the potential for cell self-renewal and differentiation. Under default parameters, the R package TCGAbiolinks [37] was used to calculate the stemness of the overall mRNA expression profile of each sample to obtain the stemness of each sample.

### Weighted correlation network analysis (WGCNA)

The top 20% of genes with the largest variance were selected from mRNA expression profiles, and the expression profiles of these genes were constructed. A systems biology analysis approach based on WGCNA was done to identify clusters of mRNA expression profiles in an unsupervised manner [38]. First, hierarchical clustering analysis was conducted using the hclust function. Then, the soft threshold power value was filtered using the pickSoftThreshold function. Candidate power values (1 to 30) were used to test the average connectivity and independence of the different modules. If the degree of independence was > 0.8, the appropriate power value was selected. Then, the highly relevant modules were further merged via mergeCloseModules function in the WGCNA R package. The highly correlated genes were used to construct correlation networks, which facilitated gene screening methods used to identify candidate biomarkers. The WGCNA R software package was then used to build a co-expression network. The minimum module size was set to 30 and was assigned a unique color for each module.

### Crosstalk analysis

Crosstalk was a hot topic in recent years. Studying the crosstalk between diseased cells may elucidate the underlying mechanism of disease [39, 40]. According to the rules, in a random background in the STRING database [41], when the interaction pairs between modules were greater than the interaction pairs in the background, this interaction is called crosstalk. The chordDiagram function of the circlize [42] package in R was used to demonstrate the interaction between modules. First, in the context of random networks, the number of interaction pairs between modules in N random networks was greater than the number of interaction pairs between modules in a real network, which was denoted as n. The formula for calculating the p value was: p = n / N (N = 1000). When p <0.05, it can be considered that the corresponding crosstalk module pairs were more significant than random. Finally, the Cytoscape software was used to display significant crosstalk in order to visually observe the complex regulatory relationships between co-expression modules.

### Functional enrichment analysis and Gene set variation analysis (GSVA)

To further identify the potential functions of genes among the ADSCs-associated modules, a Gene Ontology (GO) and Kyoto Encyclopedia of Genes and Genomes (KEGG) pathway enrichment analysis were performed. The clusterProfiler [43] package in R was employed in the enrichment analysis for the 16 modules. A p value < 0.01 was set as the cutoff criteria in the GO enrichment analysis, while a p value < 0.05 was the cutoff criteria in the KEGG enrichment analysis. In addition, the ClueGO plugin in Cytoscape [44] was used to build the GO_BP network as well as the KEGG pathway network. The GO and KEGG networks were used to reflect the relationships of GO terms according to the similarity of their related genes. A p value <0.05 was considered to be significant. GSVA [45] was used to score individual samples against the GO and KEGG pathways, and each sample received an GSVA score.
